# The Follies of Fashion

**Published:** 1882-07

**Authors:** 


### ARTICLE VII.

### "*The Follies of Fashion.*"

Just now the medical profession of England are carrying on an energetic crusade against the health destroying follies of fashion. The movement is quite universal, and seems to give promise of good accomplishments. Recently M. Fredarick Treves (*Lancet*) delivered a lecture on this subject, which promises good results, since it was a plain, intelligible, matter-of-fact exposition of the results of the deadly style of dress in vogue among fashionable women ; and was listened to by many of so-called leaders of fashion. The speaker, among other good points, said : "There is, however, no tyranny so oppressive as that of the mode. A women might as well be dead as out of the fashion. There is something mysterious in this submission, because it is perfectly well known that the rulers of fashion are not its leaders. If the facts were fully and plainly stated, the fashionable intelligence would not announce that at a particular assembly Lady Little Waist wore gorgeous apparel, of such and such material and made in such and such a style, but that Messieurs or Mesdames Snip and Stitch *exhibited a costume* on the person of Lady L. W." This statement will hold equally good in our own country. It is a dismal fact that women who desire to be fashionable seem to imagine that, to accomplish their purpose, they must resort to the most outrageous vagaries of dress, such as are calculated to seriously injure their physical welfare. The terrible habit of spending an evening in an over-heated ball-room with low neck and short sleeve dresses, and then going out into the cold night air with but little extra covering, cannot be too strongly condemned. It is a wonder that in every instance such imprudence is not rapidly followed by pneumonia. A medical journal is the proper place for the discussion of such points, since physicians possess great power and influence to mitigate the evil. The family physician, in nearly all cases, is a trusted friend and confidential adviser, in addition to his professional role. His wisdom is considered great, and his words carry much weight. If, then, physicians will earnestly combat the evils of dress, and lose no opportunity to battle against them, much good can be accomplished. For foundation stones in this warfare, let them take the fellowing points: Flannel to be always worn next the skin; clothing to be loose and suspended from the shoulders, so as not to constrict any portion of the body; shoes large and roomy; warm covering to the feet, and very little to the head. With these fundamental principles, intelligent common sense will supply the superstructure of advice in every case. Let us sincerely trust that physicians will recognize and utilize the undoubted powers they possess to reform the dress of our women, and to banish from the world much of the sickness and deformity due to our present abominable system or style of fashionable attire. To do so is a duty that physicians owe to their fellow creatures, and he who neglects to utter this little word of warning is morally speaking, criminally negligent.—*Medical and Surgical Reporter.*

